# Influence of aluminum salts on COVID-19 infected patients

**DOI:** 10.3906/sag-2009-140

**Published:** 2020-12-17

**Authors:** Ali DEMİR, İbrahim ERAYMAN, Oğuz DOĞAN, Murat KEKİLLİ

**Affiliations:** 1 Department of Gastroenterology, Meram School of Medicine, Necmettin Erbakan University, Konya Turkey; 2 Department of Infectious Diseases, Meram School of Medicine, Necmettin Erbakan University, Konya Turkey; 3 Department of Mathematics and Science Education, Department of Physics Education, Ahmet Keleşoğlu Faculty of Education, Necmettin Erbakan University, Konya Turkey; 4 Department of Gastroenterology, Faculty of Medicine, Gazi University, Ankara Turkey

**Keywords:** Coronavirus (COVID-19), aluminum salts

## Abstract

**Background/aim:**

Based on the antiviral and antibacterial properties of aluminum salts, we aimed to find out the influence of aluminum salts on COVID-19 infected patients.

**Materials and methods:**

We performed an observational retrospective cohort study which includes the patients diagnosed as COVID-19 and received aluminum salts in addition to actual treatments during hospitalization as the treatment group (Alum Group). Patients who received standard COVID-19 treatment protocols in the Infectious Diseases Clinics were included as the Control Group. Clinical findings, laboratory parameters, length of stay, survival, radiological follow-up, intensive care and mechanical ventilation needs, the presence of comorbidity, polymerase chain reaction (PCR) tests, symptoms, symptom recovery times, hospital stay times, treatment protocols, and clinical presence of pneumonia were examined in all patients. Advanced chemical composition analyzes of existing aluminum salts were also performed.

**Results:**

A total of 109 patients, 54 in the alum group and 55 in the control group, were included in the study. None of the patients in the aluminum group developed side effects due to the intake of aluminum salt. Survival status was significantly different between the two groups as there were 5 loss in the Control Group and none in the Alum Group (P = 0.023). The symptom recovery time was significantly shorter in the Alum Group; 2 (1–3) vs. 1 (1–2) days, P = 0.003. According to the paired samples analyses of the comparison between hospitalization and discharge, CRP levels significantly drops in the Alum Group (from 54.09 to 27, P = 0.001) but not in the Control Group. The drop was significantly same for the lactate dehydrogenase (LDH) and procalcitonin levels with P = 0.001.

**Conclusion:**

It has been observed that aluminum salts have beneficial effects in COVID-19 infected cases. Considering the low systemic toxicity of intermittent oral intake of aluminum salts as food supplements and the fact that pandemic control is still not achieved, the use of aluminum salts is promising.

## 1. Introduction

Corona viruses are enveloped positive-stranded RNA viruses and have hazardous effects on both humans and animals. In February 2020, the World Health Organization (WHO) designated the disease COVID-19, which stands for coronavirus disease 2019. Coronaviruses cause severe respiratory damage and failure. Due to its rapid spread and complicated infection mechanism, the whole world is faced with COVID-19 and its severe consequences.

Infection manifests a wide spectrum but the most frequent clinical presentation is pneumonia. Most of the patients have fever, cough, dyspnea, and also bilateral infiltrates on chest imaging and computerized tomography (CT) [1–4]. Patients with COVID-19 have no specific laboratory findings but most of them have lymphopenia, elevated aminotransaminase levels, elevated LDH, elevated inflammatory markers [e.g., ferritin, C-reactive protein (CRP), and erythrocyte sedimentation rate], and abnormalities in coagulation tests [3–5]. In spite of plenty of attempts and investigations, unfortunately no vaccine or curative medications have been found. Mortality rate of COVID-19 is worrisome and varies in different countries. During the COVID-19 pandemic, the global search on drugs and vaccines continues; however, a valid and effective treatment method has not been found yet. The high infectious capacity of COVID-19 and the increasing mortality rates, mostly due to respiratory failure, increase the seriousness of the situation and the need for medication. Mortality is higher in cases followed up with mechanical ventilation after lung involvement of COVID-19.

Aluminum salts, which are common in nature, are also found in drinking water in trace amounts and have been used for many years, especially in skin tanning, wound healing, bleeding control, and many other purposes. Since aluminum cannot be found pure in nature, it is in salt forms such as chloride, phosphate, sulphate, nitrate, ammonium, and acetate. Ammonium aluminum sulphate and potassium aluminum phosphate salts, which are known as blood stones in the society, are used to stop bleeding in a short time in superficial skin bleeding. There are studies on both antiviral and antibacterial therapeutic effects of aluminum salts. In addition, aluminum salts have been used safely for years as an adjuvant in vaccines.

In individuals without renal damage, the toxic effect of aluminum salts taken intermittently is not observed, and systemic toxicity findings are not expected since intestinal absorption is quite low [6–8]. In case of toxicity, the response to chelation therapies, especially desferoxamine, is quite good and results in recovery without sequelae [9,10]. Based on the antiviral and antibacterial properties of aluminum salts, it has been predicted that oral ingestion as a food supplement may have a clinically therapeutic effect against both the prognosis of primary viral disease and secondary bacterial infections that may develop after/during? viral infection in COVID-19 infected cases. In this study we aimed to find out the influence of aluminum salts on COVID-19 infected patients.

## 2. Materials and methods

We performed an observational retrospective cohort study which includes the patients diagnosed with COVID-19 between 10.03.2020 and 10.07.2020 (early pandemic) and hospitalized at Meram School of Medicine. Patients who were followed up with the diagnosis of COVID-19 in Internal Medicine Clinics received a daily salt need (2 g/day aluminum salts) as a food supplement on a voluntary basis in addition to actual treatments during hospitalization, and were included in the study as the treatment group (Alum Group). Patients who received standard COVID-19 treatment protocols in the Infectious Diseases Clinics were included as the Control Group. Aluminum sulphate (Al2(SO4)3) was used as a salt form in the study. The current study has been approved by the Necmettin Erbakan University, Meram Faculty of Medicine Ethics Committee and was performed in accordance with the rules of the ethics committee.

### 2. 1. Study design and patients

The patients in the Alum Group took a daily amount of 2 g ground aluminum salts with meals. Only adult patients (over 18 years old) were included in the study. Patients in the Alum Group have completed the COVID-19 standard treatment protocols completely and regularly until discharge and afterward. Here, the standard treatment given to the Alum Group is the same as the current and global medications applied to the Control Group patients. The study data were obtained retrospectively through investigating hospital database and patient files. All patients’ clinical findings, laboratory parameters, length of stay, survival, radiological follow-up (chest graphs and computerized tomography) and responses, presence of postdischarge sequelae, postdischarge control evaluations, possible side effect profiles, intensive care, and mechanical ventilation needs were recorded. In addition to the COVID-19 laboratory routine examinations, the presence of comorbidity, PCR tests, symptoms, symptom recovery times, hospital stay times, treatment protocols, and clinical presence of pneumonia were examined in all patients. Besides, advanced chemical composition analyzes of existing aluminum salts were also performed to compare the quality and efficacy of aluminum sulphate salts on the market and to ensure treatment standardization.

### 2. 2. Chemical analyses of aluminum salts

**Figure 1:**
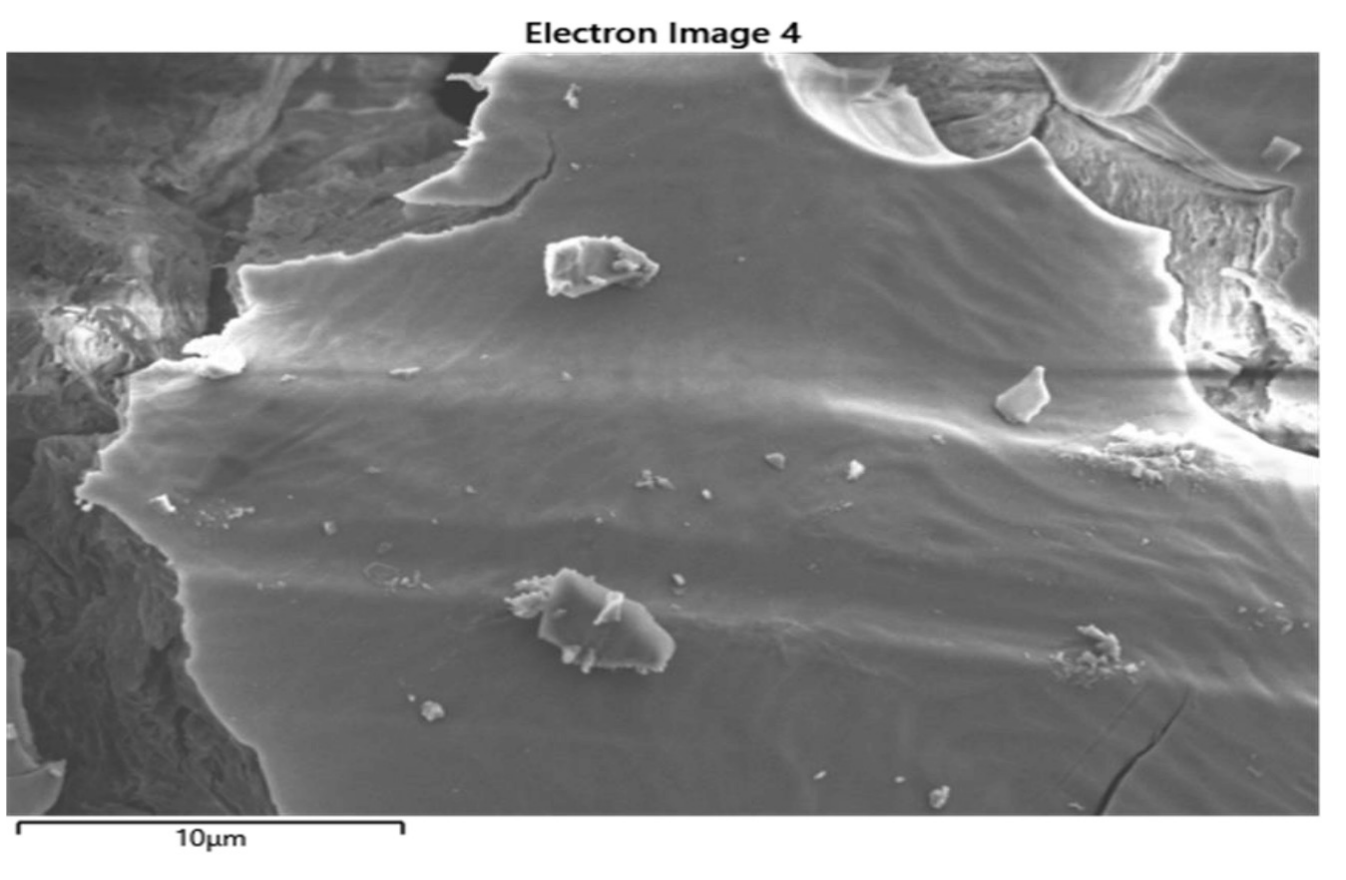
Surface imaging under 5kV voltage and 8000× magnification with ZEISS (Carl Zeiss GmbH, Oberkochen, Germany) Gemini SEM 500 Field Emission Electron Microscope (Fe-SEM) for salt with Kütahya origin.

**Figure 2:**
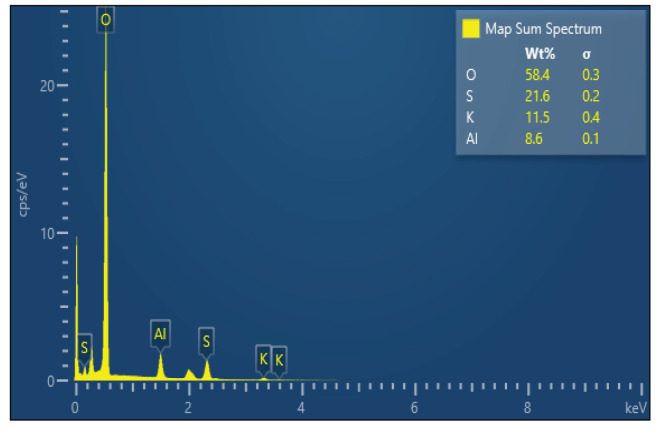
Fe-SEM-EDS spectrum and quantitative analysis result.

**Figure 3:**
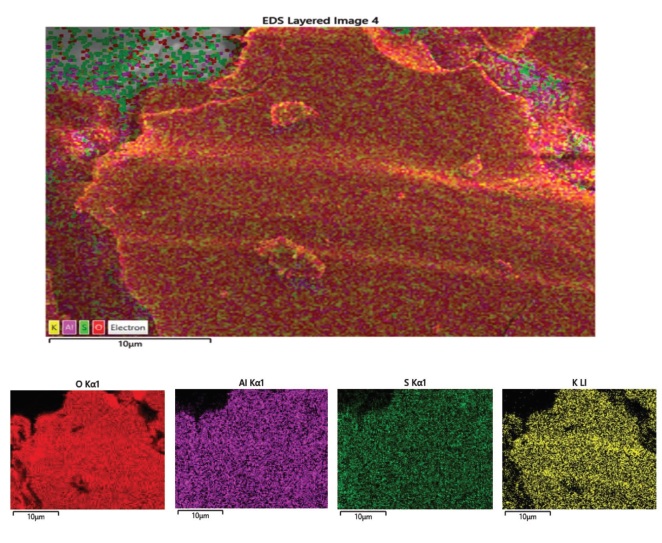
Fe-SEM quantitative analysis of elemental distribution.

**Figure 4:**
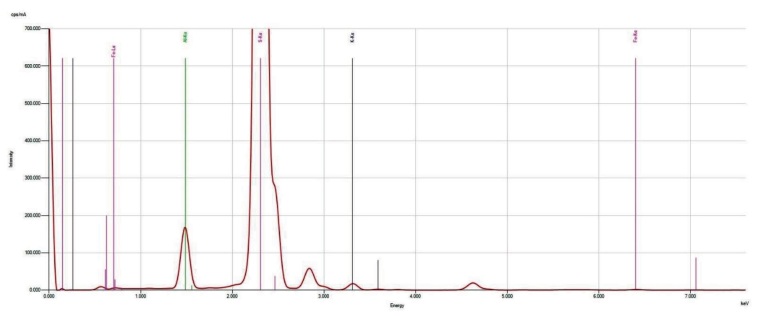
Results obtained by ED-XRF spectrum.

Since we learned that there are different types of aluminum salts sold in the market, in order to ensure standardization in the study, we used an advanced chemical analysis to make sure that the salt we use is from Kütahya/Turkey (the purest form). The characteristics of the salt sample used in the current study are as follows: certain parts of the selected sample surface are smooth, there are moderately deep cracks, there are small protrusions on the surface, and the surface has homogeneous general elemental distribution (Figure 1). In the sample, as seen in the energy dispersive spectroscopy (EDS) in Figure 2, although S, K, and Al elements have a high proportion, it is seen in Figure 3 (the spectrum is given) that there are some impurities such as Fe in addition to the elements determined as a result of Energy Dispersive X-ray Fluorescence (ED-XRF) analysis. A total of 1% change was observed between the quantitative analysis results of the sample performed with both devices (Figure 2, Table 1). This change is due to the use of the oxide structures of the elements in the calculation of ED-XRF results. Since the EDS analysis gives results for only the region where the sample is imaged, a separate analysis has been made with energy dispersive X-ray fluorescence (ED-XRF) (RIGAKU, Tokyo, Japan) spectrometer in order to make quantitative analysis of the whole sample and to determine the oxide structures of the detected elements. Additionally, quantitative analysis of elemental distribution with Fe-SEM (Field Emission Scanning Electron Microscopy) is shown in Figure 3 and Figure 4.

**Table 1:** Concentrations of the elements in the sample (ED-XRF analysis).

	SO _3_	K _2_ O	Al _2_ O _3_	Fe _2_ O _3_
	mass%	mass%	mass%	mass%
Kütahya	27.8	9.7	7.29	0.0038
Statistical error	0.0281	0.0302	0.0351	0.0004
Detection limit	0.0061	0.0016	0.022	0.0006
Quantization limit	0.0184	0.0048	0.066	0.0019

### 2. 3. Statistical analyses

Statistical analyses were performed using the IBM Statistics SPSS Software Version 23 (IBM Corp., Armonk, NY, USA). The variables were investigated using visual (histograms, probability plots) and analytical methods (Kolmogorow–Simirnov/Shapiro–Wilk test) to determine if they are normally distributed. The chi-square test or Fisher’s exact test (when chi-square test assumptions do not hold due to low expected cell counts), where appropriate, was used to compare these proportions (which proportions?) in different groups. For the variables that are not normally distributed, the Mann–Whitney U tests were conducted for comparison. The Wilcoxon test was used to compare the nonparametric two-related samples, hospitalization and discharge. The P-value less than 0.05 was considered statistically significant for all analyses.

## 3. Results

A total of 109 patients, 54 in the Alum Group and 55 in the Control Group, were included in the study. There were 25 female and 30 male patients in the Control Group, while there were 26 female and 28 male patients in the Alum Group. The median age of the Control Group was 58 (44–70), while it was 58 (44.5–70.25) for the Alum Group. None of the patients in the aluminum group developed side effects due to the use of aluminum salt. COVID-19 PCR positivity was detected in 41 (74.5%) patients in the Control Group and 13 (24.1%) in the Alum Group (statistically significant P = 0.001). The rate of comorbidity was similar in both groups: 36 (65.5%) vs. 40 (74.1%) which value belongs to which group?, P = 0.32. Survival status was significantly different between the two groups with 5 loss in the Control Group and none in the Alum Group (P = 0.023). Clinically diagnosed pneumonia presence rates were similar for both groups: 35 (63.6%) vs. 29 (53.7%) which value belongs to which group?, P = 0.24. Chest radiography and CT infiltration and Intensive Care Unit (ICU) follow-up rates were also similar in two groups (Table 2). While the durations of hospital stay were similar, the symptom recovery time was significantly shorter in the Alum Group: median 2 vs. 1 days, P = 0.003. There was no significant difference for the hospitalization duration: median 4 vs. 4 days, P = 0.92.

**Table 2:** Comparison of clinicodemographic characteristics between Control and Alum Group patients during hospitalization.

	Control (n = 55)	Alum (n = 54)	P valuea
COVID-19 PCR positivity	41 (74.5%)	13 (24.1%)	0.001
Comorbidity	36 (65.5%)	40 (74.1%)	0.32
Survival status	50 (90.9%)	54 (100%)	0.023
Pneumonia presence	35 (63.6%)	29 (53.7%)	0.24
Chest Graph infiltration	33 (60%)	25 (46.3%)	0.26
CT infiltration	32 (58.2%)	30 (55.6%)	0.37
ICU follow-up	7 (12.7%)	3 (5.6%)	0.19

CT: computerized tomography; ICU: Intensive Care Unit.aDifferences assessed using the χ2- test for categorical variables.

During the hospitalization, white blood cell, neutrophil, and platelet counts were significantly higher in the Alum Group (Table 3). While CRP levels were significantly higher in the Alum Group, the procalcitonin levels were not; 13 vs. 25.5, P = 0.036 and 0.07 vs. 0.1, P = 0.06 respectively. Fibrinogen levels were significantly higher in the Alum Group: 340 vs. 396, P = 0.025. At discharge lymphocyte levels were significantly higher in the Alum Group; 1.49 vs. 1.8, P = 0.018. CRP levels were lower in the Alum Group but was not significant; 14 vs. 13, P = 0.94 (Table 4).

**Table 3:** Comparison of laboratory parameters between Control and Alum Group patients during hospitalization (median and IQR).

	Control (n = 55)	Alum (n = 54)	P valuea
Age	58 (44–70)	58 (44.5–70.25)	0.73
Symptom recovery time	2 (1–3)	1 (1–2)	0.003
Hospitalization stay day	4 (6–9)	4 (5–8)	0.92
SO_2_	95 (92–97)	95 (92–96)	0.13
Hemoglobin (g/dL)	13.9 (12.8–14.8)	13.10 (11.87–14.52)	0.056
WBC count (x103/µL)	5.75 (4.59–7.18)	7.98 (6–10.42)	0.001
Neutrophil count (x103/µL)	3.85 (2.98–5.20)	5.89 (3.9–7.53)	0.001
Lymphocyte count (x103/µL)	1.15 (0.87–1.66)	1.22 (0.9–2.05)	0.23
Platelet count (x103/µL)	191 (145–225)	224 (185–279)	0.002
Creatinine (mg/dL)	0.8 (1–1.24)	0.93 (0.8–1.2)	0.63
AST (IU/dL)	21 16–33)	19 (14–31)	0.31
ALT (IU/dL)	16 (12–25)	16 (11–29)	0.65
CRP	13 (6.7–37)	25.5 (10.3–67.75)	0.036
Sedimentation	26 (14.5–49)	27.5 (15.75–58.5)	0.28
LDH	232 (198–290)	250 (195–313)	0.41
Procalcitonin	0.07 (0.05–0.12)	0.1 (0.05–0.27)	0.07
Ferritin	133 (63–256)	156 (86–649)	0.1
D-DIMER	176 (93–368)	252 (132–650)	0.1
Fibrinogen	340 (292–382)	396 (287–547)	0.025
INR	1 (1–1)	1 (0.94–1.2)	0.15

SO_2_: saturation of oxygen.aDifferences assessed using the Mann–Whitney U test for numerical variables.

**Table 4:** Comparison of laboratory parameters between Control and Alum Group patients at discharge (median and IQR).

	Control (n = 55)	Alum (n = 54)	P valuea
SO_2_	96 (95–97)	96 (95–97)	0.51
WBC count (x103/µL)	5.7 (4.9–7)	7.1 (5.67–8.7)	0.004
Neutrophil count (x103/µL)	3.45 (2.9–4.2)	4.14 (3.44–5.28)	0.014
Lymphocyte count (x103/µL)	1.49 (0.97–1.98)	1.8 (1.28–2.66)	0.018
Platelet count (x103/µL)	209 (175–274)	241 (196–330)	0.05
Creatinine (mg/dL)	0.94 (0.79–1.1)	0.89 (0.72–1.18)	0.74
AST (IU/dL)	24 (17–40)	18 (13–32)	0.023
ALT (IU/dL)	21 (15–40)	18 (11–36)	0.07
CRP	14 (5–29)	13 (5–39)	0.94
Sedimentation	33 (12–59)	39 (19–56)	0.57
LDH	230 (185–305)	217 (168–279)	0.46
Procalcitonin	0.06 (0.04–0.09)	0.07 (0.04–0.17)	0.63
Ferritin	223 (100–428)	225 (92–625)	0.9
D-DIMER	187 (95–376)	269 (127–569)	0.21
Fibrinogen	366 (271–432)	382 (273–469)	0.64
INR	1 (1–1)	1 (0.9–1)	0.81

SO_2_: saturation of oxygen.aDifferences assessed using the Mann–Whitney U test for numerical variables.

**Figure 5:**
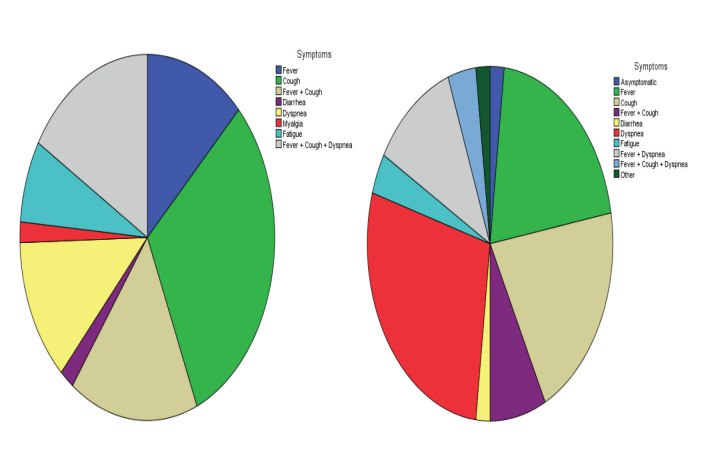
Distribution of symptoms at hospitalization for Control (A) and Alum (B) Groups.

**Figure 6:**
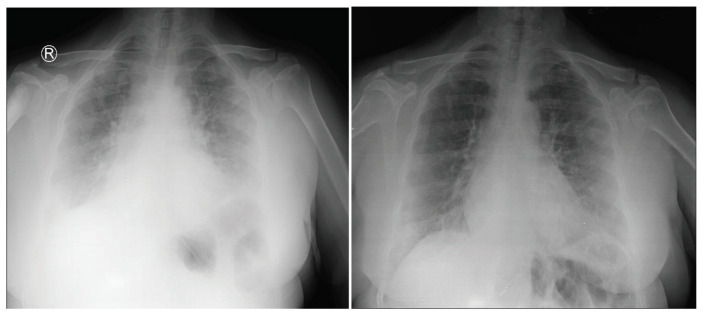
The chest radiographs of a patient who used aluminum salts, taken at 2 days interval.

According to the comparison of the paired samples analyses during the hospitalization and after discharge, CRP levels were found to drop significantly in the Alum Group (from 54.09 to 27, P = 0.001) but not in the Control Group (Table 5). The drop was significantly same for the LDH and procalcitonin levels with P = 0.001 (Table 6). The distributions of symptoms at hospitalization are depicted in the Figure 5. In order to evaluate the clinical effect of aluminum salts, the chest radiographs of a patient who used aluminum salts were taken at 2 days interval (Figure 6).

**Table 5:** Paired samples comparison of laboratory parameters for Control Group between hospitalization and discharge (mean ±SD).

	Hospitalization	Discharge	P valuea
SO_2_	93.85 ± 4.65	95.69 ± 2.63	0.001
WBC count (×10^3^/µL)	6.12 ± 2.36	6.71 ± 3.61	0.83
Neutrophil count (×10^3^/µL)	5.57 ± 9.71	4.56 ± 3.61	0.29
Lymphocyte count (×10^3^/µL)	1.29 ± 0.59	1.54 ± 0.64	0.001
Platelet count (×10^3^/µL)	189.4 ± 56.6	227.7 ± 101.1	0.028
Creatinine (mg/dL)	1.08 ± 0.40	1.08 ± 0.58	0.06
AST (IU/dL)	76.7 ± 358	41.2 ± 51.7	0.51
ALT (IU/dL)	62.8 ± 305	54.2 ± 134.5	0.003
CRP	29.57 ± 37.79	32.26 ± 52.83	0.49
Sedimentation	33.1 ± 27.8	38.4 ± 26	0.58
LDH	260.2 ± 98.8	262.2 ± 123.7	0.72
Procalcitonin	0.1 ± 0.1	3.34 ± 18.88	0.44
Ferritin	218.6 ± 310.1	685.8 ± 1570	0.001
D-DIMER	449.7 ± 829.1	483.2 ± 717.5	0.53
Fibrinogen	355.3 ± 110.8	365.7 ± 106.9	0.96
INR	1.01 ± 0.9	1.05 ± 0.16	0.18

SO_2_, saturation of oxygen.a Differences assessed using the Wilcoxon Signed Ranks Test for paired samples.

**Table 6:** Paired samples comparison of laboratory parameters for Alum Group between hospitalization and discharge (mean ±SD).

	Hospitalization	Discharge	P valuea
SO_2_	93.45 ± 3.53	95.70 ± 1.84	0.001
WBC count (×10^3^/µL)	8.44 ± 3.50	7.71 ± 3.17	0.02
Neutrophil count (×10^3^/µL)	6.40 ± 3.41	4.71 ± 2.27	0.001
Lymphocyte count (×10^3^/µL)	1.49 ± 0.79	2.06 ± 1.14	0.001
Platelet count (×10^3^/µL)	232.1 ± 80.1	269.5 ± 110	0.055
Creatinine (mg/dL)	1.32 ± 1.27	1.31 ± 1.27	0.06
AST (IU/dL)	40.8 ± 81.4	36.3 ± 87.2	0.59
ALT (IU/dL)	25.1 ±25.8	30.3 ±39.5	0.37
CRP	54.09 ±65.71	27 ±36.45	0.001
Sedimentation	40.2 ±30.6	42.8 ±29.8	0.11
LDH	319.4 ±271.4	302.7 ±472.8	0.001
Procalcitonin	7.16 ±41.2	0.31 ±1.05	0.001
Ferritin	838.3 ±3216	371.4 ±346.8	0.86
D-DIMER	630.3 ±847.1	608.2 ±1213	0.21
Fibrinogen	431.4 ±175.7	382.1 ±125.8	0.11
INR	1.12 ±0.36	1.11 ±0.43	0.36

SO_2_: saturation of oxygen.aDifferences assessed using the Wilcoxon signed ranks test for paired samples.

## 4. Discussion

Based on the antiviral and antibacterial properties of aluminum salts, it has been predicted that oral ingestion as a food supplement may have a clinically therapeutic effect against both the prognosis of primary viral disease and bacterial infections that may develop secondary to viral infection in COVID-19 infected cases. We retrospectively found that aluminum salts have beneficial effects on both clinical and laboratory findings in COVID-19 infected patients. While the effects observed in favor of loss-free survival and decrease in symptom recovery time, it is also pleasing that no side effects are observed due to aluminum salts.

Based on the results of the chemical in terms of the purity and efficacy of the aluminum salts, we believe that treatment standardization is achieved. In addition, this study should also be taken into account in prospective studies to be carried out with aluminum salts. Since the period during which the study was conducted was an early pandemic, the PCR test could only be performed on patients who were hospitalized at that time, 48 to 72 h after hospitalization. The patients in the Alum Group started taking aluminum salts at the time of hospitalization, and they used aluminum salts for at least 48 h before the PCR test was performed. In our study, we believe that the low PCR-test positivity in the Alum Group developed due to the possible antiviral activity of aluminum salts within this delay period. One of the most striking results of this study is that there is no loss of life in the Alum Group; however, it would not be right to make an assertive comment with a retrospective study conducted with a limited number of patients. In evaluation of the therapeutic responses of patients to aluminum salts, we found that there was no inequality? in terms of pneumonia during hospitalization by clinical and radiological evaluations carried out in order to evaluate respiratory system involvement that most frequently caused mortality in COVID-19 patients. However, we found that the time needed for the improvement of the symptoms, which we considered clinically as a criterion for recovery, was significantly shorter in the Alum Group. In terms of the inflammation burden, we found that the white blood cell (WBC), neutrophil, CRP, and procalcitonin levels checked during hospitalization were higher in the Alum Group than in the Control Group, but were lower than in the Control Group after discharge. In addition, after the evaluation of parameters with prognostic value such as the presence of lymphopenia and high fibrinogen in the follow-up of COVID-19, we found that although the levels of admission fibrinogen in the Alum Group were higher than the Control Group, they were lower after discharge, and similarly, they had higher lymphocyte levels after discharge.

There is not enough data in the literature regarding the therapeutic use of aluminum salts in humans. There are also many toxicity studies. Many adverse events and complications, especially Alzheimer’s disease, dementia, and kidney failure are mentioned due to chronic and high dose intake of aluminum salts. However, the beneficial effects of aluminum salts such as antiviral and antibacterial effects are also known. Foot-and-mouth disease (FMD) or hoof-and-mouth disease (HMD) is an u3014 and sometimes fatal u3015 u3016 that affects u3017 u3018, including domestic and wild bovid [11,12]. Aphthovirus is a u3019 genus of the family u301a. Aphthovirus infect u301b, and include the causative agent of u301c. u301d (FMDV) is the prototypic member of the genus Aphthovirus [13]. In our country, animals suffering from foot and mouth disease are treated quite successfully with the topical use of aluminum salts. As it is known, aluminum salts have been used safely for years as an adjuvant in vaccines. In a study conducted with aluminum chloride salts, it was observed that the adsorption of the virus to the filter increased [14]. Skin wraps impregnated with aluminum acetate have been found to have an effective antibacterial activity [15]. In a study performed with rabbit alveolar macrophages, it has been shown that in vitro aluminum chloride hydroxide salt increases the phagocytic effect [16]. In a recently published study, it was stated that aluminum ammonium salts have a very effective topical disinfectant effect, especially against nosocomial infectious agents, which occurs within seconds [17].

In our study, no side effects and toxicity were observed due to the intake of daily 2 g of aluminum salts of the patients with meals. In addition, since the patients took aluminum salts with meals, there were no tolerance and adaptation problems. Aluminum salts are very easy to procure and are very cheap. As we found in our study, the recovery time for the symptoms is quite short. Although the patients in the Alum Group received aluminum salts in addition to the current COVID-19 treatments and treatments were applied in different protocols, no drug interaction and complications were detected in any patient. In our study, we think that better results can be obtained with higher doses because patients took aluminum salts at the lowest dose (as much as the daily salt need) for safety precautions.

Due to the retrospective nature of the study, a limited number of cases were examined with inadequate methodology. Due to the limitation in PCR test in the early pandemic period, most patients could only be tested after the treatment was started, which is a factor that reduces the reliability of the test. Another negative factor is that patient data are not recorded at the desired standard. However, despite all these negativities, the current study is the first and only study done in this concept so far.

As a result, it has been observed that aluminum salts have beneficial effects both clinically and in the laboratory on COVID-19 infected cases. Considering the low systemic toxicity of intermittent oral intake of aluminum salts as food supplements and the fact that pandemic control is still not achieved, the use of aluminum salts is promising. In the future, through the prospective studies with more patients, we can make clearer comments on the contribution of aluminum salts to both treatment and prophylaxis of COVID-19 infected patients.

## Informed Consent

Written informed consents were obtained from patients.

## Ethics Committee Approval

Necmettin Erbakan University, Meram School of Medicine Ethics Committee – 2020/2781 – 07.08.2020.

## Author Contributions

Concept - 1, 2, 3, 4; Design - 1, 2, 3, 4; Supervision - 1, 2; Resources - None; Materials – 1, 3; Data Collection and/or Processing - 1, 2, 3, 4; Analysis and/or Interpretation–1, 2; Literature Search–1, 2; Writing Manuscript –1; Critical Review - 1, 2, 3, 4; Other – None.
